# Surgical treatment of ankle instability in children with os subfibulare

**DOI:** 10.1007/s00402-023-04905-y

**Published:** 2023-05-29

**Authors:** Marcin Zgoda, Matthew C. A. Arnold

**Affiliations:** Department of Trauma and Orthopaedic Surgery, University Hospital Crosshouse, Kilmarnock Road, Crosshouse, KA2 0BE Scotland

**Keywords:** Os subfibulare, Ankle instability, Children, Ligament reconstruction

## Abstract

**Introduction:**

Ankle instability in children due to soft tissue injury usually resolves after non-operative treatment. However, some children and adolescents with chronic instability require surgical treatment. A rarer cause of developing ankle instability is injury to the ligament complex in the presence of os subfibulare, an accessory bone inferior to the lateral malleolus. The aim of this study was to assess the results of operative management of chronic ankle instability in children with os subfibulare.

**Materials and methods:**

16 children with os subfibulare and chronic ankle instability who failed non-operative treatment were enrolled prospectively into the study. One child was lost to follow-up and excluded from analysis. The mean age at the time of the surgery was 14 years and 2 months (range 9.5–17 years). The mean follow-up time was 43.2 months (range 28–48 months). Surgical treatment in all cases involved removal of os subfibulare and a modified Broström-Gould lateral complex reconstruction with anchors. Ankle status was assessed before and after surgery with The 100 mm Visual Analogue Scale and Foot and Ankle Outcome Score questionnaire.

**Results:**

The mean Foot and Ankle Outcome Score improved from 66.8 to 92.3 (*p* < 0.001). Pain level improved from 67.1 preoperatively to 12.7 (*p* < 0.001). All children reported improvement in their ankle stability. There was one case of scar hypersensitivity that improved during observation and one superficial wound infection that resolved with oral antibiotics. One child reported intermittent pain without symptoms of instability following another injury.

**Conclusions:**

Ankle joint sprain with associated injury to os subfibulare complex can lead to chronic instability in children. If conservative management fails, then surgical treatment with modified Broström-Gould technique and excision of accessory bone is a safe and reliable method.

## Introduction

Os subfibulare is a rounded bony fragment inferior and anterior to the lateral malleolus with a prevalence of 1–2% [1, 2]. The aetiology of os subfibulare remains unclear. One theory suggests that os subfibulare is a secondary ossification centre that fails to fuse with the fibula. When present, the secondary ossification centre of the distal fibula usually appears on radiographs at approximately 7 years of age and fuses with the rest of the fibula at age 15–17 [3]. More recent evidence suggests that os subfibulare may result from trauma with the separation of non-united tip of the lateral malleolus [4]. Os subfibulare is often an incidental finding on foot and ankle radiographs taken due to trauma, however, some children with os subfibulare may complain of localised lateral ankle pain and symptoms of functional instability. In most cases, symptoms often resolve after non-operative management, but some patients require surgical treatment. The surgical management of chronic ankle instability in the presence of os subfibulare in cases where physiotherapy fails is still unclear [5].

Various surgical techniques have been reported to address ankle instability in the presence of os subfibulare in adult and adolescent populations. One of the techniques is an anterior talo-fibular ligament (ATFL) reconstruction using a Broström or Broström–Gould technique with the excision of os subfibulare [6, 7]. Other techniques include open or arthroscopic fusion of the os subfibulare to the distal fibula with a screw [8]. In the presence of pain without instability, simple excision of the accessory bone has demonstrated good results [9]. The current orthopaedic literature consists of only small series or case reports focused on the paediatric and adolescent population treated by lateral ligament reconstruction with the use of anchors for ankle instability in the presence of os subfibulare [10, 11].

The aim of our study was to assess mid-term results of surgical management of a chronic lateral ankle instability in the presence of os subfibulare with the Broström-Gould lateral ligament reconstruction following failure of conservative management.

## Methods

### Patient selection

Study approval was granted by the local NHS Research Ethic Board (REB) prior to recruiting patients (Registration Number 35/2011/REB). 26 children with os subfibulare were referred to our institution between January 2012 and December 2018 with ankle pain and instability. 9 children from this group had already failed physiotherapy prior to referral. The remaining 17 children who did not have any rehabilitation were initially referred for guided physiotherapy. The physiotherapy programme involved focused strengthening and stretching exercises of peroneal muscles, proprioception exercises including single leg standing and weight shifting wobble board training, hip strengthening and gradual return to normal activities including sports. The physiotherapy programme was the same for the group of 9 children referred straight to physiotherapy and the other 17 who were first seen by the orthopaedic department and later referred to physiotherapy. Physiotherapy took place at the same physiotherapy department, by the same physiotherapists and utilised the same rehabilitation protocol. Due to having similar physiotherapy treatment, the subgroups were not compared.

In 8 cases, the physiotherapy programme was successful, and surgery was not required. 2 other children did not attend physiotherapy or their review appointment. The remaining 7 children plus 9 whom physiotherapy had previously failed qualified for surgical treatment.

**Fig. 1:**
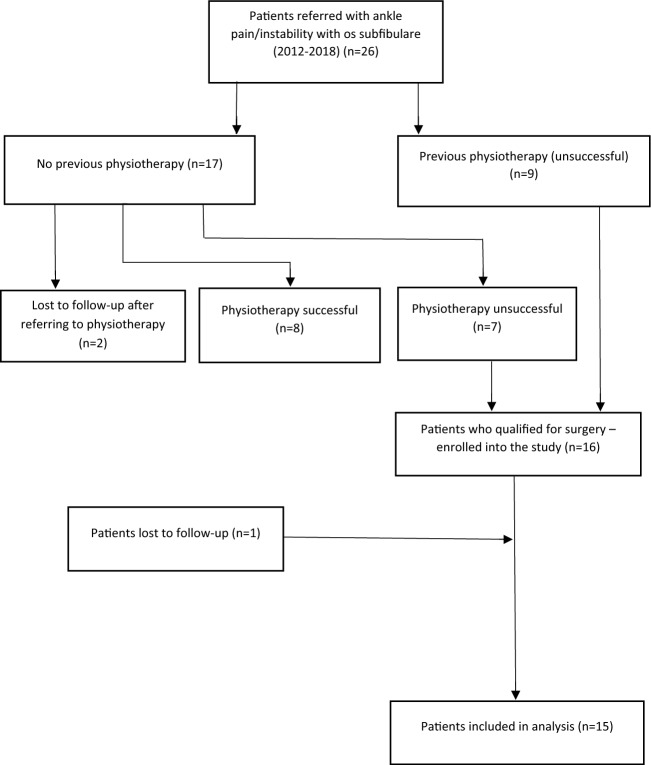
Flowchart of patients’ selection process

Inclusion criteria for enrolment into the study were: age below 18, pain and symptoms of functional instability, unsuccessful physiotherapy programme, presence of os subfibulare on ankle radiographs; willingness to participate in a study and informed consent given by the child and parents/carers. Exclusion criteria were previous bony injury, chronic conditions of the limb which may affect functional status of the ankle (e.g. talipies equino-varus; neuromuscular conditions), connective tissue disease with joint hypermobility (e.g. Ehlers–Danlos syndrome) and previous surgeries to the foot or ankle. The minimum follow-up period was 24 months. The study was designed to follow-up children for 48 months post-operatively, or longer if clinically indicated. Patient selection process is presented in Fig. 1. In total, 16 children were prospectively enrolled into the study group, however, one patient was lost to follow-up and hence 15 patients were included in analysis. There were 6 boys and 9 girls. The mean age at the time of the surgery was 14 years and 2 months (range 9 years and 8 months–17 years). The left ankle was affected in 8 children and right in 7 children. 11 children reported a history of trauma to the ankle. All children reported ankle pain localised over os subfibulare and on the lateral aspect of the ankle joint and reported symptoms of functional instability. Instability of the ankle was confirmed on a clinical assessment with positive anterior drawer test and apprehension on forceful supination of the foot. All patients had conventional radiographs of the ankle (AP, mortise and lateral views) and MRI scan of the ankle. All MRI scans showed a single loose os subfibulare except 1 patient who had a fibrous connection between the os subfibulare and fibula, and 1 patient who had an os subfibulare consisting of two ossicles.

### Surgical technique

All surgeries were carried out under general anaesthetic with the use of a pneumatic tourniquet. Fluoroscopy was not routinely used. An anteriorly curved 4–5 cm long incision was made below the lateral malleolus centred over os subfibulare and anterior tibiofibular ligament (ATFL) complex. The os subfulare was exposed and the anterior talo-fibular and calcaneo-fibular ligaments were identified. The os subfibulare was rounded to an oval bone with the proximal end of ATFL attached to the ossicle. Os subfibulare was removed and the tip of the lateral malleolus was refreshed for better ligament healing. ATFL and if required calcaneo-navicular ligament was reattached to the tip of the lateral malleolus using two anchors (Mini QUICKANCHOR Plus, DePuy Mitek) with non-absorbable Ethibond Excel sutures (Ethicon; Johnson&Johnson) as described by Broström [12]. These were attached to the distal portion of the fibula at the site or in direct proximity to the bed from the avulsed os subfibulare. This area is usually 1 to 1.5 cm distally from the distal fibular growth plate. The ligament was then reinforced with extensor tendon retinacular flap using Gould technique [13]. Care was taken to avoid the physis, and there was no incidence of growth plate injury or growth arrest during follow-up. All repairs felt stable after fixation, and no other additional stabilisation was required. There was moderate swelling and thickening of the peroneal tendons observed in two children and mild erosion of the talus that required debridement in one patient, but no other pathology was observed that required additional surgical intervention. The wound was closed in a standard fashion using subcuticular absorbable sutures. Drains were not used. Post-operatively, the leg was immobilised in a below knee non-weight bearing cast for four weeks followed by a walker orthosis for a further 2 weeks. Active and supported range of motion, strengthening and proprioception exercises were commenced at that time.

### Assessment of outcome

Symptoms and function of the ankle was assessed using the 100 mm Visual Analogue Scale (VAS), with 0 being no pain and 100 being the worst pain. The Foot and Ankle Outcome Score questionnaire (FAOS) was also completed before and after surgery. FAOS is a 42-item questionnaire assessing patient-relevant outcomes in 5 separate subscales (Pain, Other Symptoms, Activities of Daily Living, Sport and Recreation Function, Foot- and Ankle-Related Quality of Life) and is regarded as a useful tool for the evaluation of patient-relevant outcomes related to ankle reconstruction [14]. Children were followed up at 6 weeks, 12 weeks, 6 months and on a yearly basis thereafter. Results were analysed using SPSS (version 28.0.1.0) and *p* value < 0.05 considered statistically significant.

## Results

**Fig. 2:**
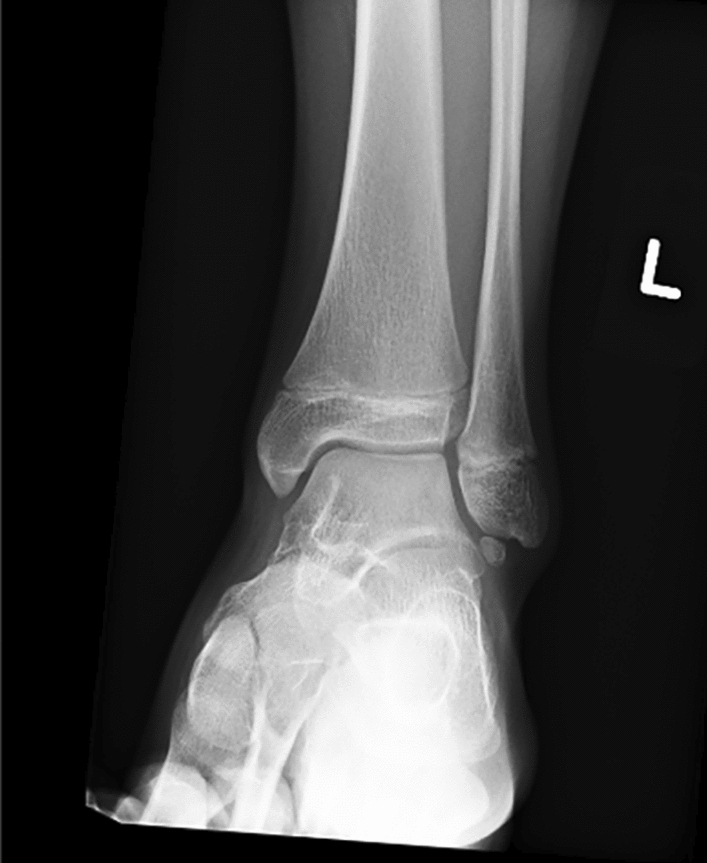
Ankle radiograph with os subfibulare

**Fig. 3:**
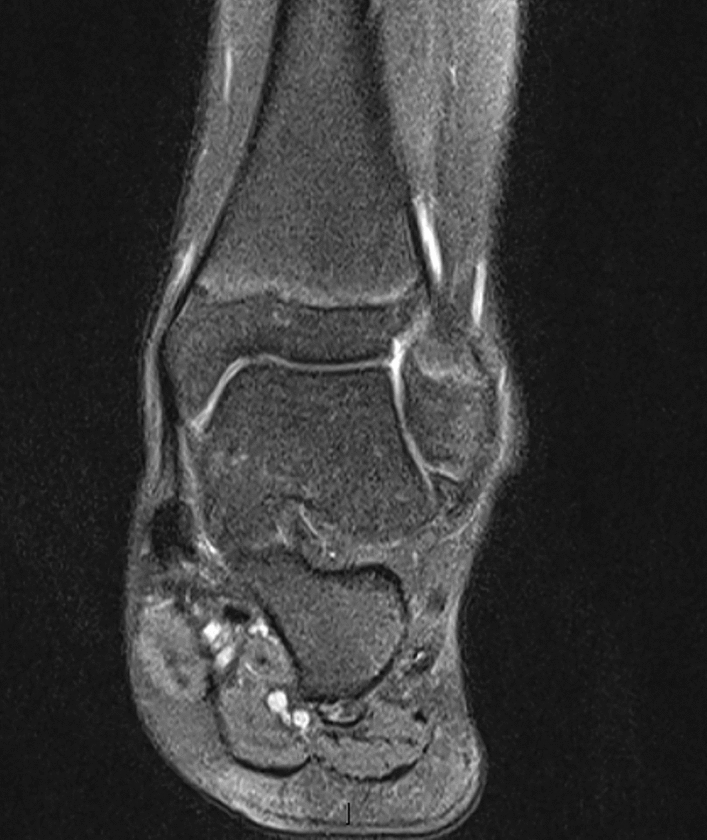
MRI scan of the ankle with os subfibulare-ligamentous complex

**Fig. 4:**
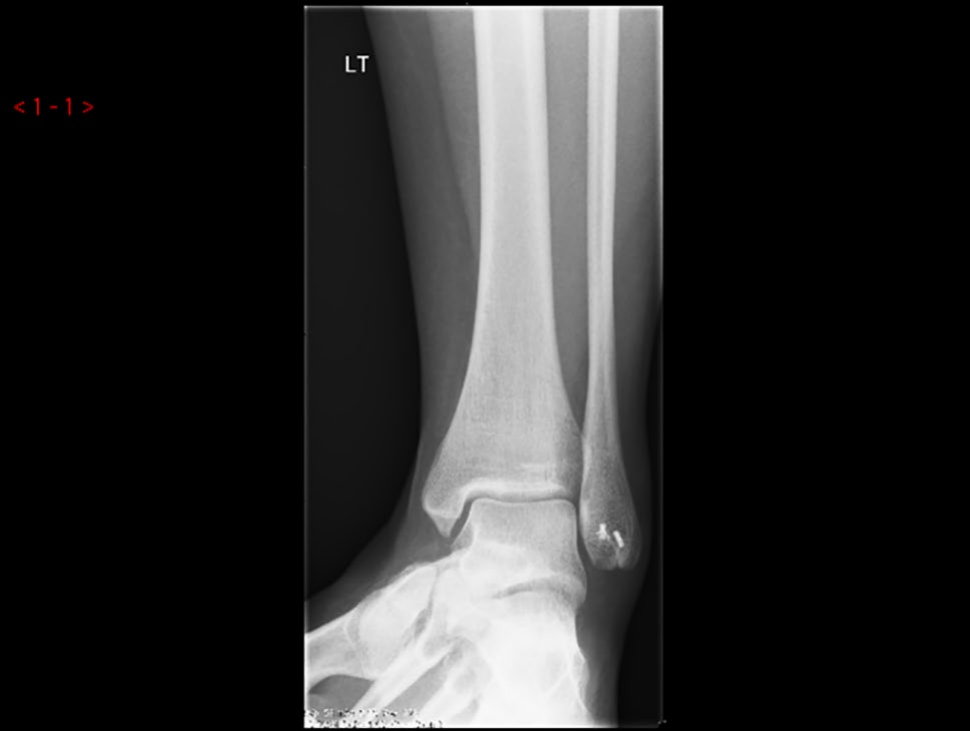
Post-operative X-ray: resection of os subfibulare and repair of the ATFL with anchors

**Fig. 5:**
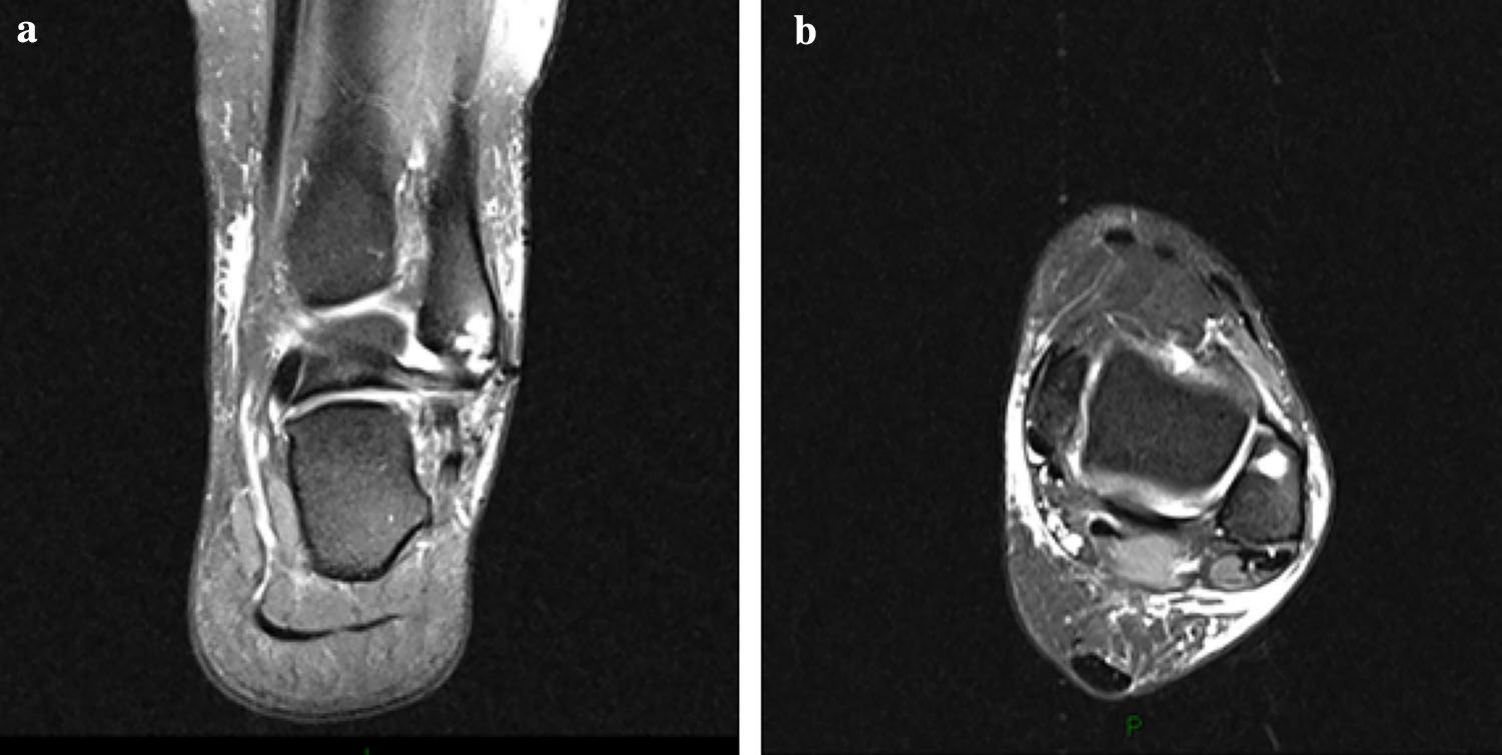
Post-operative MRI scan with anchors (**a** coronal view, **b** axial view)

15 children completed at least 24 months of follow-up and were include in the study, patient characteristics are summarised in Table 1. Most procedures were performed as day cases (*n* = 13). 2 children were kept overnight in the hospital for pain management and physiotherapy. The mean hospital stay was 1.1 day. The mean follow-up was 43.2 months (range 28 months to 48 months; SD-7.59). There was one case of scar hypersensitivity that improved during observation. 1 patient developed superficial post-operative wound infection that responded to oral antibiotics and healed without sequel. 1 child reported intermittent pain without symptoms of instability following another injury. All children reported improvement in ankle stability. The mean Foot and Ankle Outcome Score improved from the mean 66.8 (range 54–81; SD-7.48) preoperatively to 92.3 (range 84–98; SD-4.02) at the latest follow-up (*p* < 0.001). The mean VAS pain level before surgery and at the latest follow-up visit improved from 67.1 (range 47 to 80; SD-10.09) preoperatively to 12.7 (range 0–31; SD-9.44) (*p* < 0.001). The average time to return to recreational sport or to physical education at school was 12.5 weeks (range 9–18 weeks; SD-2.5). 6 out of eight children returned to their sporting activities at a competitive level following surgery (Figs. 2, 3, 4, 5).

**Table 1:** Patient characteristics and outcomes

Patient	Age at surgery (years)	Gender	History of trauma	Follow-up (months)	FAOS-pre op	FAOS- post op	VAS pre-op	VAS follow-up	Return to PE (weeks)
1	14.2	F	Y	48	68	90	74	12	12
2	12.8	F	Y	48	72	92	53	8	14
3	9.8	M	N	36	60	84	80	26	10
4	15.7	F	Y	48	58	89	78	8	16
5	14.1	M	N	48	81	98	47	5	15
6	13	M	Y	36	54	93	76	22	12
7	14.9	M	Y	24	70	96	68	10	10
8	15.5	F	Y	48	67	95	59	0	13
9	17	M	Y	48	64	92	70	16	14
10	13.2	F	N	48	72	95	64	12	11
11	15.3	F	Y	36	59	86	76	24	18
12	14.8	F	N	48	78	94	64	4	12
13	15.7	F	Y	48	63	90	78	31	9
14	12.8	F	Y	36	71	98	63	0	10
15	13	M	Y	48	65	92	57	13	12
Mean	**14.1**			**43.2**	**66.8**	**92**	**67.1**	**12.7**	**12.5**

## Discussion

Our results indicate that the modified Broström-Gould reconstruction of ATFL complex combined with the excision of os subfibulare provide excellent mid-term results. Pain and instability improved following surgery in all patients and every child resumed their sporting activities. This is in agreement with previous studies [6, 7]. In the retrospective study of Phil et al., 23 children with ankle instability and os subfibulare were treated at a mean of 10.4 years with resection of os subfibulare and repair of ATFL with modified Broström technique with drill holes made through the distal fibula. At the mean follow-up of 4.5 years, all but one patient were symptom free. The mean pain level decreased from 7.2 to 2.1 at the latest follow-up. The mean Foot and Ankle Outcome Score at the last follow-up visit was 91 (range 86–99). Similar excellent results were achieved by Kubo et al. [7]. Authors carried out a retrospective study of 31 patients with chronic ankle instability and the presence of os subfibulare who had undergone resection of the ossicle with lateral ligament reconstruction using suture anchors. Clinical outcomes were evaluated by American Orthopaedic Foot and Ankle Society Ankle-Hindfoot Scale (AOFAS) and Karlsson-Peterson ankle function scores. The mean follow-up was 40.7 months. Mean AOFAS score increased from 66.3 (range 62–77) preoperatively to 96.5 (range 87–100) at the final follow-up. Mean Karlsson-Peterson score increased significantly from 51.7 (range 47–70) preoperatively to 95.3 (range 80–100) at final follow-up.

In the absence of objective instability, simple excision of os subfibulare with subsequent cast immobilisation and physiotherapy gives satisfactory results in the study of Moukoko et al. [9]. The authors compared 17 patients with functional instability of the ankle and os subfibulare, but without objective laxity of the ankle joint (resection group) with 19 patients who received only physiotherapy (control group). At the mean of 4 years and 4 months, a significant improvement of the American Orthopaedic Foot and Ankle Society score was observed and was significantly higher in the resection group with a mean gain of 31 points versus 7 points in the control group. Monden et al. utilised arthroscopic excision of symptomatic os subfibulare without instability of the ankle in 19 patients with an average age of 17.6 years [15]. Results were assessed using Japanese Society for Surgery of the Foot (JSSF) ankle/hindfoot scales. The mean score improved form 77.6 ± 2.6 points preoperatively to 97.2 ± 5.2 points post-operatively (*p* < 0.01). In our series, all children included in the study had clinical, objective lateral instability of the ankle joint with positive anterior drawer test and ankle varus test. Lateral ligament reconstruction was carried out in all patients from our study with satisfactory results. We, therefore, advocate this technique rather that simple os subfibulare excision when lateral ankle instability is present.

Kose et al. presented a case of os subfibulare injury that was treated successfully with two screw fixation in a 19-year-old woman [16]. Other authors observed that the time to achieve union may take a few months and do not recommend this technique [5, 17]. This technique may be useful in adult patients or in those where the fragment is large enough.

Our study has some limitations. The patient group is relatively small but comparable to other studies. The incidence of os subfibulare and associated persistent instability that do not respond to physiotherapy is a rare phenomenon in paediatric and adolescent population. All patients were included prospectively into the study and were treated with the same surgical technique and post-operative rehabilitation protocol. Another limitation of the study is the lack of control group for comparison—children who were not treated surgically. All children from our study had a course of physiotherapy prior to surgery and only those whom conservative treatment failed were qualified for surgical reconstruction and enrolled into our study. We regarded physiotherapy as the first modality of treatment for functional instability in children, therefore, we did not want to compare directly the results of operative and non-operative management [18]. Longer follow-up may give clearer answer about the long term effect on the function and stability of the ankle joint following ligament reconstruction with Broström-Gould technique and excision of os subfibulare. Finally, ultrasound has been shown to be a useful tool for assessing ankle instability [19]. In this study, clinical examination and symptoms of instability in addition to MRI was used to assess the ankle joint, but the use of ultrasound may have added further information.

## Conclusion

Ankle joint sprain with associated injury to os subfibulare complex can lead to chronic instability in children. If conservative management fails, surgical treatment with modified Broström-Gould technique and excision of accessory bone is a safe and reliable method to restore ankle stability and allow children to return to their normal activities. Further studies are needed to assess long term results of lateral ligament reconstruction with excision of os subfibulare.
